# Correspondence

**Published:** 1851-01

**Authors:** E. Locke

**Affiliations:** Hamilton


Editor Dental Register:—In looking over a very valuable medical journal entitled "Braithwaite's Retrospect," I find the following, which, if practical, must be of immense value to the profession. My object in communicating it to you is, that it may be spread abroad through the columns of the Register, so that some gentleman may prepare the article and report its worth to the profession in the Mississippi Valley.

The article to which I allude is entitled as follows:

A New Preparation for Stopping Teeth, by J. S. Davenport, Esq., of Bloomsbury.—[Mr. Davenport gives the following recipe:]—Pour a small quantity of collodion on a plate of glazed surface; allow it to evaporate till it acquires the consistence of thick paste, or, in more familiar language, a pill consistence. Let the cavity of the tooth be well dried, and quickly fill with the paste; in the course of a few minutes it will be hard and fit for mastication. Very slight pressure is required, and being of a vegetable nature somewhat analogous to the tooth itself, will resist the action of the vegetable juices, and remain colorless. [Mr. D. prepared his collodion in a manner different from that usually adopted. He says:]—My recipe differs from the usual process in three respects, viz.: an increased proportion of sulphuric acid, a longer period for maceration and an additional quantity of absolute alcohol. By the first and second alterations I obtain a more perfectly soluble cotton; by the latter, the solution is considerably modified in its power of contraction which is desirable for the aforesaid purpose.

The chemical manipulation is extremely disagreeable, the nitrous acid fumes are very abundant, consequently highly irritating to the respiratory organs.

Great care is necessary in well washing the cotton, also, a moderate heat in drying it.

Recipe:—Take Nitrate of potash, 4 lbs.
Sulphuric acid, 8 "
Carded cotton wool, 8 oz.

Mix the nitrate and acid in a glazed vessel, add the cotton and constantly agitate with a glass rod for half an hour, then wash the cotton thoroughly in cold water, so that no trace of acid should be perceptible to test paper, then dry carefully, and the result will be a very soluble gum cotton.

Then take one ounce of the cotton, rectified sulphuric ether, 16 ounces fluid; when dissolved add one ounce absolute alcohol.

Allow the solution to stand twenty-four hours, and the collodion will be ready for use.

I also find in the same journal the following, which should be prepared by some chemist or druggist and put into market, that the profession may judge of its value:

NEW REMEDY FOR TOOTH-ACHE, BY DR. BUSHNAN.

This remedy never fails.

This is CARVACROL, ($C_{26}H_{18}O_2 = HOC_{26}H_{17}O$)

Carvacrol, according to Professor Schweirtzer, is formed by the action of potassa iodine, or hydrated phosphoric acid upon oleum carui, Ol. Thymi, and, according to Claus by the action of iodine upon camphor. Schweitzer has shown that the product from camphor is the same as that obtained from the oil of caraway.

Prepared as follows:

1. Ol. Caraway is to be distilled with hydrated phosphoric acid. The liquid that passes over is to be poured back into the retort until it no longer retains the smell of the caraway. The *Carvacrol* separates itself in the form of an oil from the phosphoric acid.

2. In the same way a saturated solution of iodine must be distilled with oil of caraway until no more hydriodic acid is formed. The red mass which remains in the retort must be operated upon by potash. The yellow solution is to be distilled. Carvene ( $C_{10}H_8$ ) passes over, and the carvacral remains. It is to be purified by re-distillation.

3. Equal parts of camphor and iodine are to be rubbed together and distilled until no red vapor is given off.

The black mass remaining in the retort contains carbon, resin, camphine, colophine, iodine and *carvacrol;* that in the receiver, camphene, colophene, *carvacrol*, and a little iodine and hydriodic acid, which, on standing, separates into two layers. The upper layer is to be distilled, and at 180°, camphene passes over while colophine and *carvacrol* remain. This is to be acted on by potash. The *carvacrol* may be obtained from the alkaline solution by the action of an acid and by distillation over purified lime.

Carvacrol is an oily liquid very similar to creosote, with a very unpleasant smell and strong taste. Applied on a piece of cotton to a decayed and painful tooth, *it gives immediate relief.*

As the above recipes have found their way into Journals which do not often fall into the hands of dentists, I deem it proper for them to make their appearance in a more conspicuous place. You will therefore let them appear in your Journal with such criticisms as you deem proper.*

Yours, for light and knowledge,

E. LOCKE.

HAMILTON, Nov. 12, 1850.

---

*The preparation for filling teeth may be about as useful as "Hill's Soft Fillings." The tooth is not a vegetable substance. We think that either gold or tin is more durable. All preparaions for curing 'tooth-ache' will succeed only in certain cases. Extracttion is the only cretain remedy. Every dentist who understands the correct pathology of this disease will understand why inflammation of the dental nerve and of the periosteum will require different treatment.—ED.

